# The Performance of a New Multidimensional Frailty Index in Comparison to the Frailty Phenotype to Assess Frailty in People Living with HIV 50 Years of Age and Older in an Urban HIV Clinic

**DOI:** 10.33696/aids.7.058

**Published:** 2025

**Authors:** Uzoamaka A. Eke, Katie Wasserstein, Carolyn Susman, Ahizechukwu C. Eke, Kareshma Mohanty, Sarah Schmalzle, Nicole Viviano, Jennifer D Hoffmann, Neha S. Pandit, Robyn Palmeiro, Judith Lee, Alice S Ryan, Kristen A. Stafford, Ann Gruber-Baldini

**Affiliations:** 1 Division of Clinical Care and Research, Institute of Human Virology, University of Maryland School of Medicine, Maryland, USA; 2 Penn State College of Medicine, Pennsylvania, USA; 3 Perelman School of Medicine, University of Pennsylvania, Pennsylvania, USA; 4 Johns Hopkins School of Medicine, Maryland, USA; 5 University of Maryland Baltimore, Maryland, USA; 6 University of Maryland School of Pharmacy, Maryland, USA; 7 Baltimore Geriatric Research, Education, and Clinical Center (GRECC), The Veterans Affairs Maryland Health Care System, Maryland, USA; 8 Department of Medicine, University of Maryland School of Medicine, Maryland, USA; 9 Center for International Health, Education, and Biosecurity (CIHEB), Institute of Human Virology, University of Maryland School of Medicine, Maryland, USA; 10 Department of Epidemiology and Public Health, University of Maryland School of Medicine, Maryland, USA

**Keywords:** Frailty, HIV and Aging, Frailty and HIV, Frailty index, Frailty assessments, AIDS

## Abstract

**Background:**

Frailty is increasingly recognized in older people living with HIV (PLWH), but optimal diagnostics are yet to be determined. Frailty indices (FI) represent an accumulation of health deficits shown to correlate better with mortality and adverse effects of aging than the frailty phenotype or chronological age.

**Methods:**

This is a retrospective cohort study of frailty assessments in PLWH aged ≥ 50 years in a multidisciplinary urban HIV clinic. Frailty was assessed using Frailty Phenotype (FP) and a new 40-variable clinical composite FI derived from routine clinical and laboratory data (CCFI). CCFI scores were categorized into robust (≤ 0.15), pre-frail (>0.15-0.4), and frail (>0.4). CCFI frailty and its association with frailty-related factors were analyzed using logistic regression.

**Results:**

The 165 participants were mostly black (94%) and male (56%), with median age 59 years (IQR 55-63), CD4 count 606 cells/μl (IQR 393-873), and 78% had HIV viral load ≤ 40 copies/ml. 70% had multimorbidity, 38% falls, 25% poor cognition, and 24% polypharmacy. By FP, 2% were frail, 65% prefrail, and 33% robust. By CCFI, 26% were frail, 67% prefrail, and 7% robust (range 0.08-0.57; mean 0.34 ±0.11). For FP categorized as robust, prefrail and frail, the mean CCFI was 0.31 ± 0.1, 0.35 ± 0.11 and 0.38 ± 0.08 respectively (P=0.06). Cognition (OR 3.64, p=0.003), falls (OR 5.09, p<0.001), polypharmacy of 6-9 medications (OR 3.07, p=0.03) and ≥ 10 medications (OR 4.25, p=0.009) and >3 comorbidities (OR 3.06, p=0.03) were associated with CCFI frailty, adjusted for age and sex.

**Conclusion:**

The majority of older PLWH were pre-frail or frail. The CCFI identified more patients as frail and had significant clinical associations compared to FP.

## Introduction

Half of all people living with HIV (PLWH) in the United States currently are aged 50 years and above and this number is projected to rise to 70% by the year 2030 [1,2]. Anti-retroviral therapy (ART) has dramatically increased life expectancy after diagnosis among PLWH; however, with this increased lifespan, the burden of non-AIDS defining conditions among PLWH has also increased [3,4]. Cardiovascular, cognitive, and functional deficits, bone loss as well as frailty occur at an earlier age in PLWH, compared to those without HIV, thereby increasing their risk for morbidity and mortality [5,6].

Frailty is a clinical syndrome characterized by a decline in physiological and functional reserve, leading to increased vulnerability and inability to handle usual life stressors, which results in adverse outcomes such as falls, disability, institutionalization, and death [6]. Frailty is increasingly recognized in older adults with HIV (OAWH) despite sufficient viral suppression and occurs up to two decades earlier (~age 40 years) compared to those without HIV, with a prevalence of 5-30% depending on the frailty measure [7-9]. In PLWH and OAWH, frailty has been attributed to a combination of factors including systemic immune activation involving a complex interplay of pro-inflammatory cytokines and lymphocyte dysregulation from HIV replication, chronic inflammation, coinfections, multimorbidity, polypharmacy, and ART toxicity [6,10,11].

Multiple approaches have been used to describe, define, and diagnose frailty but the optimal method in PLWH is yet to be determined. The Fried frailty phenotype (FP) is one of the most widely used descriptions of frailty, comprising 1) weight loss of 10 lb. or more over a year; 2) self-reported exhaustion; 3) slow walking speed; 4) weakness as determined by grip strength, and 5) low physical activity. The presence of three or more of these is a diagnosis of frailty, whereas individuals with one or two elements are diagnosed as pre-frail [12]. However, it has been shown that the definition of frailty transcends a phenotype, leading to other methods of identifying and characterizing frailty, such as the use of a multidimensional frailty index (FI) [13,14].

A FI is typically a collection of clinical conditions and laboratory values, from which a frailty score is calculated as a ratio of the total number of health deficits or conditions, to the total number of variables assessed. Hence, FI represents an accumulation of deficits over time, which eventually leads to outcomes of frailty such as falls, disability, institutionalization, and mortality [13,15]. Several variables in different combinations have been studied and validated by several studies as an effective measure of frailty. The original FI derived by Rockwood *et al.* included 100 variables, but studies show that an index of 30-40 variables was as effective, valid, and less cumbersome [16,17]. Although both Fried’s FP and FI have been used and validated in PLWH, FI correlates with mortality and other adverse outcomes of frailty to a greater degree than FP and chronological age [13,15,18-21]. Moreso, the prevalence of frailty in PLWH also depends on the tool of measure, with a lower prevalence (~10%) obtained using the FP, versus a higher prevalence (~30%) reported with the FI, which captures some of those categorized as pre-frail by the FP [7,8,22]. FI has not been studied substantially in a predominant African American population and some of the indices have comprised variables and markers that are not easily obtainable in routine patient care.

In this study, a new 40-variable clinical composite FI construct (CCFI) was derived as a combination of variables that have been used in other validated frailty indices. CCFI, comprising real world variables that are used in routine patient care, was compared to the Fried frailty phenotype (FP) as a tool for diagnosing frailty, to assess for association between both measures and clinical outcomes in OAWH.

## Methods: Retrospective Cohort Study

### Setting and patient population

The Strengthening Therapeutic Resources in Older Adults Aging with HIV (STRONG) study incorporated standardized geriatric assessments in PLWH ≥ 50 years of age at the Together Healing, Reaching, Inspiring, to achieve Victory over Illness and Embrace life (THRIVE) clinic at the University of Maryland Medical Center, Midtown Campus, Baltimore, MD. THRIVE is a multidisciplinary center that serves over 2500 PLWH in Baltimore City. Clients aged 50 years and older presenting for routine care were informed of the study. Consenting clients were enrolled and underwent the geriatric assessments between November 2019 to February 2022. The study was approved by the University of Maryland, Baltimore Institutional Review Board.

### Frailty phenotype (FP)

FP frailty was determined by trained clinic staff as part of the geriatric assessments by using an adapted assessment from Kunadan *et al.* [23] which indicated frailty if 3 of the 5 following criteria were met: 1) self-reported unintentional weight loss in the last year or a BMI of less 18.5 kg/m^2^; 2) low physical energy in the last 4 weeks; 3) low physical activity; 4) poor grip strength measured in kilograms in the dominant hand, by obtaining the average of 3 readings using a digital hand-held dynamometer; 5) slow walking speed – measured using the Timed Up and Go (TUG) test over a distance of 10 feet [24]. FP scores of 0, 1-2, and ≥ 3 out of 5 criteria represented robust, pre-frail, and frail, respectively.

### Clinical composite frailty index (CCFI)

A new 40-variable multidimensional FI construct (CCFI) was generated, comprising demographic, clinical, geriatric, and behavioral risk factors, HIV parameters, and laboratory values that are used in routine HIV care, which have been used in other validated frailty indices. CCFI components were selected based on expert opinion for content validity, which means that the variables were acquired age and health related deficits that encompassed a range of physiologic systems, which must be reasonable and sensible in the field in which they are being applied. The original construct had 50 variables. Variables with >5% missing values were excluded, leaving 40. None of the variables was rare (<1%) or too common (>80%). All values were obtained from EMR chart review. Most recent values within 6 months of the study visit were used for laboratory markers. The clinical, geriatric, and behavioral risk factors were identified through review of patients’ HIV visit progress notes and utilizing EMR search bar for each term, which locates the term in all notes, laboratory, and imaging fields. If the patient had an ever diagnosis or history of the condition as a problem documented in the chart or mentioned in the notes at any point in time, it was recorded as present. Polypharmacy was determined during the STRONG study visit based on all the medications that the patient was prescribed at that point in time, including over the counter medications. CCFI was calculated as a fraction of the deficits present in each patient to the total variables that comprised the index. For example, if 10 deficits are counted from a total of 40 variables that comprise the index, the CCFI will be 10/40 = 0.25. If the value for any variable in the index was not available for a patient, then the denominator total will be less than variable. For instance, if 10 deficits are counted and data on 2 variables are missing from a 40-variable index, then the CCFI will be 10/38= 0.26. CCFI scores of ≤ 0.15, >0.15-0.4 and >0.4 were categorized as robust, pre-frail, and frail respectively, based on proposed cut points identified using stratum specific likelihood ratios from other studies [25]. The variables used and their definitions are shown in Table 1.

### Validation of the CCFI

CCFI was validated using a 3-pronged approach, comparable to what has been used in other studies [15,17,26,27].

1. Expert opinion for content validity, which means that the contents of the index are acquired age and health related deficits that encompass a range of physiologic systems, which must be reasonable and sensible in the field in which they are being applied.
2. Construct validity was demonstrated by including at least 30 variables, utilizing variables that have been included in validated frailty indices in PLWH, and demonstrating the consistent submaximal limit of 2/3 of deficits or 99 % of study participants have FI <0.7 (The maximum FI in our study was 0.57).
3. Predictive validity, which was demonstrated by the significant association of our CCFI frailty with frailty related outcomes, such as falls, poor cognition, and multimorbidity.

### Data analyses

Descriptive analyses were performed for all data collected. CCFI and FP were compared with respect to frailty prevalence for the study population and different categories using Person’s chi square for categorical variables. Analysis of variance was used to compare the mean CCFI for the FP categories (robust, pre-frail, and frail). Multivariate logistic regression models adjusted for age and sex were used to examine the association between CCFI frailty and each of the following factors: poor cognition history, falls, polypharmacy (≥10 medications), multimorbidity (≥2 history of hypertension, diabetes, hyperlipidemia or chronic kidney disease, defined as eGFR <60 ml/min), history of disability, CD4 count and thrombocytopenia.

## Results

Of the 184 participants enrolled from November 2019 to February 2022, 165 patients completed the assessments. Majority (94%) were Black and male (56%), with median age 59 years (IQR 55-63) and median duration of HIV infection was 21 years (IQR 16-29). 78% were virally suppressed (HIV RNA ≤40 copies/ml), median CD4 count was 606 cells/μl (IQR 393-873), and hepatitis C co-infection prevalence was 52%. Fifty-three percent had multimorbidity (≥ 2 comorbidities), 38% had falls, 25% had poor cognition history, 24% had polypharmacy and 32% did not complete high school (Table 2).

**Figure 1.**
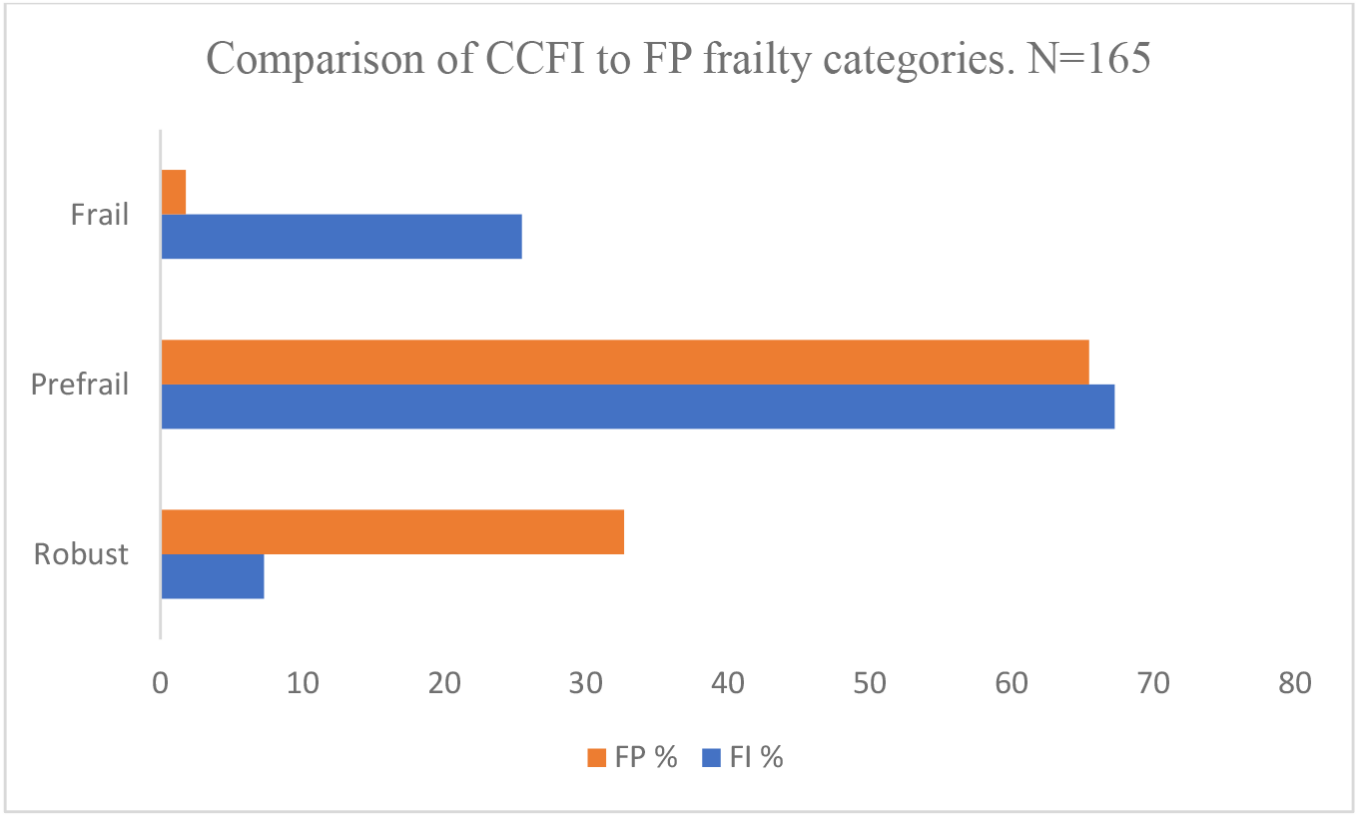
Comparison of CCFI to FP Frailty Categories. N=165.

**Figure 2.**
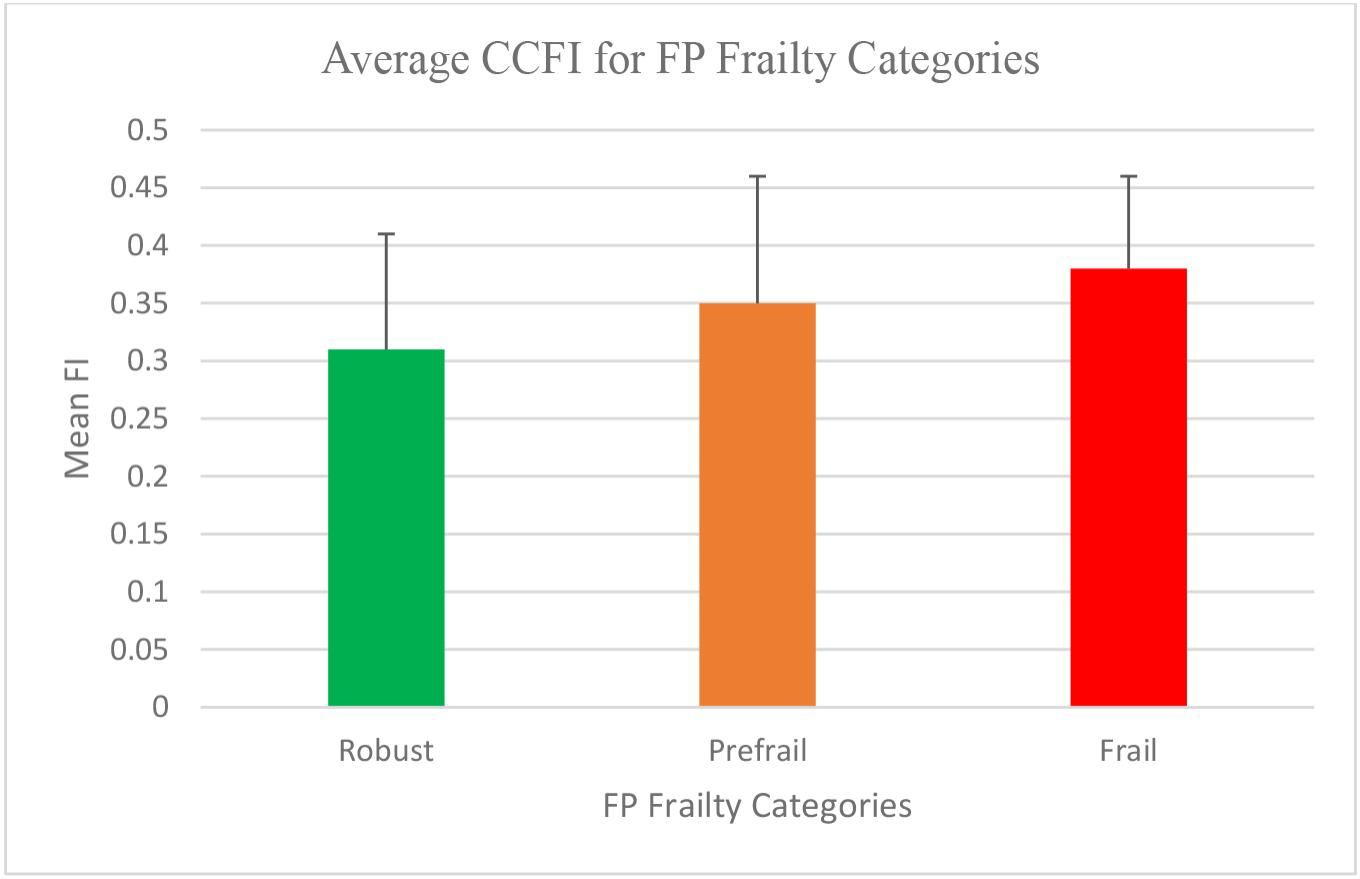
Average CCFI for FP Frailty Categories. P=0.06 (Analysis of variance).

The predominant FP characteristic was weakness (50.9%), followed by shrinking (20.6%) and low physical activity (17%) (Supplementary Figure 1). Using FP, 2% were frail, 65% prefrail, and 33% robust (Figure 1). CCFI ranged from 0.08-0.57; mean 0.34 ± 0.11. Using CCFI, 26% were frail, 67% prefrail, and 7% robust (Figure 1). There was a significant difference between the CCFI frailty results for the following patient characteristics: mean age, employment, smoking and injection drug use (IDU) history, HIV infection duration, polypharmacy, history of falls, and history of poor cognition. No difference was seen in the FP frailty categories for any patient characteristics (Table 2). For FP categorized as robust, prefrail, and frail, the mean FI was 0.31 ± 0.1, 0.35 ± 0.11 and 0.38 ± 0.08 respectively (P=0.06) (Figure 2).

Poor cognition (OR 3.64, p=0.003), falls (OR 5.09, p<0.001), Polypharmacy of 6-9 medications, OR 3.07, p=0.03 and 10 medications, OR 4.25, p=0.009), multimorbidity (>3 comorbidities OR 3.06, p=0.03), disability (OR 3.80, p=0.004) and thrombocytopenia (OR 5.72, p=0.001) were among the factors associated with CCFI frailty in multivariate logistic regression analysis, after adjusting for age and sex (Table 3). All these factors except multimorbidity and disability (OR 2.31, p=.06) remained significant using a modified 39-item CCFI that excluded each of these factors (Supplementary Table 1). In the case of multimorbidity, a 37-item index was used that excluded hypertension, diabetes and GFR, which are the components of multimorbidity that are present in the index. There was no significant association of CCFI with CD4 count, HIV viral load, and BMI.

## Discussion

The clinical composite frailty index (CCFI) identified 13 times as more patients as frail (26%) and had significant associations with frailty related outcomes, compared to Fried’s Frailty Phenotype (FP), which identified only 2% of the population as frail. Because only 3 patients were diagnosed as frail using FP, we were unable to determine any FP frailty associated factors because a logistic regression analysis could not be performed. On the other hand, both measures identified about 65% of the study participants as pre-frail.

The varying prevalence of frailty using different methods has been shown in other studies, which have identified a higher prevalence of frailty using previous FI compared with the FP. A recent meta-analysis of 1.7 million participants, 50 years and older, pooled from 62 countries showed frailty prevalence of 11-13% from phenotype measures versus 22-26% for frailty index measures [9]. FI has also been shown to be more sensitive in predicting frailty associated adverse outcomes, including mortality than FP [28]. Although we could not conduct a regression analysis with FP in our study due to the small sample number of frail patients by FP, our CCFI was also sensitive in predicting frailty associated adverse outcomes like other FI studies. This advantage of FI over FP is attributed to its ability to recognize frailty on a continuous scale that extends beyond physical characteristics, ranking patients based on the number of deficits present, from the most robust (fittest) to the most vulnerable (frailest) [15] . In this regard, FI could be utilized and optimized for earlier identification and potential intervention for frail patients, who would otherwise be undiagnosed if only FP was used. However, these studies are not specific to PLWH, and the number of Black participants is not clearly elucidated, and appear at best to be underrepresented, which presents the need for more FI studies in Blacks and in PLWH.

Our study identified only 3 patients (2%) as frail using FP. This prevalence is lower than data from other studies among PLWH 50 years and older from various demographics, using FP, where frailty prevalence has ranged from 8-14.2% overall, and even higher in women (17%) [7,29,30]. Data on physical frailty among Black PLWH is sparse, with limited data from African cohorts reporting a 9-19% prevalence, while a study among HIV-negative Medicare enrollees ≥ 65 years of age identified frailty in 23% of Blacks, compared to 14% of Whites [31-33]. Our low FP frailty prevalence may be attributed to our small sample size, differences in demographics and heterogeneity in the determination of FP criteria used in different studies. For instance, the cut off used for the timed up and go test ranges from 10-19 seconds, and the latter was used in our study, as adapted from Kunadan *et al.* [23,34-36]. The wide gap in frailty prevalence in our study between FP and CCFI suggests that FP alone may not be an adequate assessment in PLWH, and perhaps Black PLWH in particular. On the other hand, 2/3 of our patients were identified as pre-frail, using both FP (65%) and CCFI (67%). Other studies in PLWH have identified 40-54% as pre-frail [29,30]. The large proportion of this at-risk, pre-frail population points to the work needed to mitigate or reverse the progression to frailty, especially given the accelerated aging phenotype of PLWH. Weakness as determined by grip strength was the most prevalent (50%) FP category in our study. There is variation in the predominant FP categories among PLWH cohorts but exhaustion (37-46%) and low physical activity (20-40%) were more common in some Black predominant PLWH cohorts >40 years of age [31,32].

Our CCFI construct had significant associations with several important frailty related adverse factors including poor cognition, falls, polypharmacy, and multimorbidity. The definition of polypharmacy in PLWH is not standardized. Our study shows that taking 6-9 or ≥ 10 medications was a predictor of frailty. Although the association of polypharmacy with phenotypic frailty has been described in PLWH and non-HIV-infected populations, its association with FI frailty is limited, as it is for the other significant factors above. The association of cognitive impairment and the spectra of HIV-associated neurocognitive disorders with frailty and other geriatric syndromes has been described in OAWH, remains a huge burden (35-50%), and is associated with mortality, despite viral suppression and immune recovery [31,37]. Our study identified poor cognition in about a quarter (24.8%), which may be an underrepresentation, possibly limited by chart review. Physical frailty has been linked to injurious falls and mortality in several cohorts of PLWH [38]. Guraldi’s FI showed an association with falls in a European OAWH cohort. The current study provides an opportunity to show this important association using our CCFI in a predominantly African American OAWH population. Multimorbidity was no longer a significant association after the FI was modified by removing the variables that comprised it. Our definition of multimorbidity was limited to only patients with a documented history of hypertension, diabetes, hyperlipidemia or CKD to reduce heterogeneity. Nonetheless, over 50% of our patients had multimorbidity which is double the 22-25% prevalence that is reported in other studies among PLWH [39]. The significant association of thrombocytopenia with our CCFI is interesting. This could imply that thrombocytopenia may be a potential marker for frailty in OAWH and warrants further study.

Our CCFI did not show an association with CD4 count as has been described in other FP and FI studies [7,18,25]. This may stem from the fact that only 10 (6%) of our patients had a CD4 count <200 cells/ul. Other significant clinical associations with CCFI frailty in our study including disability, IDU, smoking, and hepatitis C co-infection have also been identified in other studies [40,41]. However, when the CCFI was modified to a 39-item index by removing each of these factors, there was no longer a significant association with these factors. Nonetheless, the concept of a frailty index as an accumulation of deficits over time makes it important to include these factors especially pertaining to their role and potential impact in our PLWH population.

Studies using FI to diagnose frailty in PLWH are sparse and several methods have been used to validate the index. Our CCFI was validated using a 3-pronged approach as described in the methods, comparable to what has been used in other studies [15,17,26,27]. The mean CCFI index for each FP category in our study progressively increased in robust, prefrail, and frail, as shown in other studies [25]. Although this did not reach statistical significance (P=0.06), this trend suggests a correlation between CCFI and FP that may have been significant if the sample size was larger, which contributes to the validity of our index. In our patient population, FI may be a more accurate tool to identify frail patients. In this modern era of machine learning and artificial intelligence, the CCFI variables could be automated by the EMR to generate a frailty score for each patient immediately either upon request or intuitively, removing the need for physical assessments that may be more cumbersome to perform in PLWH. The application of CCFI in HIV care in this manner may provide a timely diagnosis of frailty and recognition of patients at risk for frailty, which may likely reduce the barrier to diagnosis and time to intervention in PLWH.

Our study has several strengths including the novelty of the CCFI, the assessment in an inner city predominantly African American patient population, and its cross-sectional, real-world approach where the patients underwent clinical assessments as they would during a regular clinic visit. The CCFI was derived from practical, clinical, behavioral, and laboratory values that are obtained during routine HIV care. Moreover, it was compared to an already established, widely applied, and phenotypic index, which has been validated in the HIV population. Nonetheless, there are several limitations worth noting. One weakness is that the clinical aspects of the CCFI were obtained from chart review, implying that the data was limited to what clinicians documented in the EMR. Only three patients in the study were FP frail, which limited the ability to perform analyses with FP frailty. In addition, since the majority of our patients were Black, which correctly reflects our predominantly Black population in the city of Baltimore, our findings may not be applicable to other demographics. Nonetheless, the burden of HIV infection is pronounced in the Black population, which make up over 40% of PLWH in the United States [42].

## Conclusion

The need for accurate and timely diagnosis of frailty in HIV will continue to be an important innovative stride as PLWH continue to age and the understanding of frailty continues to evolve. The frailty index is a promising alternative, especially if automated and included as part of the routine evaluation and care of people living with HIV. Our clinical composite frailty index construct identified more patients as frail in our HIV population and was associated with frailty related outcomes compared to the frailty phenotype. As only 3 people were identified as frail using FP, this suggests that FP may fail to identify individuals who though functionally frail with accumulated health deficits, do not exhibit the physical features of frailty. In this situation, the frailty index may provide a more sensitive and clinically useful measure of frailty in PLWH. Both frailty measures identified over 2/3 of patients as pre-frail, representing an at-risk population that could be intervened upon to mitigate and prevent frailty. More research is needed to optimize the CCFI, including automation and applicability to different demographics and larger populations, where it could be studied as a stand-alone frailty diagnostic tool in people living with HIV.

## Supplementary Material

JAHT-25-058-Supplementary-Files

## Figures and Tables

**Table 1.** Health Variables Included in the 40-item Clinical Composite Frailty Index.

Variable Category	No.	Variable	Deficit Description (All obtained from chart review)
Demographics			
	1	Education level	Less than complete high school education
	2	Employment	Unemployed
Comorbidities			
	3	Hypertension	Hypertension history
	4	Diabetes mellitus	Diabetes history
	5	Chronic kidney disease	GFR <60 ml/min
	6	CVD	History of CVA, TIA, MI, or PVD
	7	Hepatitis C	Positive hepatitis C antibody
	8	Liver disease	Liver cirrhosis, MASLD
	9	COPD	COPD history
	10	Congestive heart failure	History of congestive heart failure
	11	Malignancy	History of any malignancy
HIV parameters			
	12	HIV RNA	>40 copies/ ml
	13	CD4 count	<200 cells/ul
	14	HIV infection diagnosis duration (years)	>20 years since HIV diagnosis
	15	Nonadherence to antiretroviral therapy	Documented history of non- adherence to antiretroviral therapy
Geriatric factors/Function			
	16	Cognition/memory difficulty	History of cognition or memory difficulty documented in the EMR
	17	Disability	History of documentation of disability in the EMR
	18	Fall/s	History of falls
	19	Difficulty walking	History of difficulty walking
	20	Hospitalization	History of any hospitalization in the past year
	21	History of infection	History of any documented infection in the past year
	22	History of surgery	History of any surgery in the operating room in the past year
	23	Depression and/or anxiety	History of depression and/or anxiety
	24	Chronic pain	History of chronic pain
	25	Polypharmacy	10 or more prescribed medications
Behavioral and other risk factors			
	26	Tobacco smoking history	Ever smoked regularly
	27	IDU history	Ever used injection drugs
	28	ASCVD risk score	≥ 20%
	29	BMI	<18 or >25
Laboratory values			
	30	Total cholesterol	>200 mg/dl
	31	LDL	>100 mg/dl
	32	HDL	<40 mg/dl
	33	Triglycerides	>150 mg/dl
	34	Transaminases	ALT and AST above upper limit of normal
	35	Hemoglobin	<11 g/dl Female <12 g/dl Male
	36	Platelet	<150 cells/ul
	37	WBC	<4,500 /ul
	38	Albuminuria or proteinuria	Positive on urine dipstick
	39	Serum albumin	<3.5 g/dl
	40	Serum vitamin D	<20 ng/ml

**Abbreviations:** GFR: Glomerular Filtration Rate; CVD: Cardiovascular Disease; COPD: Chronic Pulmonary Disease; CVA: Cerebrovascular Disease; TIA: Transient Ischemic Attack; MI: Myocardial Infarction; PVD: Peripheral Vascular Disease; MASLD: Metabolic Dysfunction-Associated Steatotic Liver Disease; IDU: Injection Drug Use; ASCVD: Atherosclerotic Cardiovascular Disease; BMI: Body Mass Index; LDL: Low Density Lipoprotein; HDL: High Density Lipoprotein; WBC: White Blood Cell

**Table 2.** Characteristics of the study Cohort by FP versus CCFI Frailty Status.

	FP Frailty				CCFI Frailty				
Characteristics	Total N=165 (%)	Robust N=54 (%)	Pre-frail N=108 (%)	Frail N=3 (%)	*P*	Robust N=12 (%)	Prefrail N=111 (%)	Frail N=42 (%)	*P*
**Mean Age years, SD**	59.2 ± 5.6	59.4 ± 5.7	59.0 ± 5.5	57.7 ± 4.5	.89	55.3 ± 4.2	58.9 ± 5.5	60.8 ± 5.4	**<.01**
50-54	36 (21.8)	13 (24.1)	22 (20.4)	1 (33.3)	.75	6 (50.0)	26 (23.4)	4 (9.5)	.09
55-59	55 (33.3)	14 (25.9)	40 (37.4)	1 (33.3)		4 (33.3)	36 (32.4)	15 (35.7)	
60-64	46 (27.9)	15 (27.8)	30 (27.8)	1 (33.3)		2 (16.7)	30 (27.0)	14 (33.3)	
≥65	28 (17)	12 (22.2)	16 (14.8)	0 (0)		0 (0)	19 (17.1)	9 (21.4)	
**Race**									
White	10 (6.1)	3 (5.6)	7 (6.5)	0 (0.0)	.88	2 (16.7)	5 (4.5)	3 (7.1)	.23
Black	155 (94.0)	51 (94.4)	101 (93.5)	3 (100.0)		10 (83.3)	106 (95.5)	39 (92.9)	
**Sex at birth**									
Male	92(55.8)	34 (63.0)	59 (54.6)	0 (0.0)	.08	7 (58.3)	62 (55.9)	24 (57.1)	.98
Female	73(44.2)	20 (37.0)	49 (45.4)	3 (100.0)		5 (41.7)	49 (44.1)	18 (42.9)	
**Educational attainment**									
≥12 years	113 (68.5)	39 (72.2)	73 (67.6)	2 (66.7)	.83	11 (91.7)	78 (70.3)	24 (57.1)	.06
<12 years	52 (31.5)	15 (27.8)	35 (32.4)	1 (33.3)		1 (8.3)	33 (29.7)	18 (42.9)	
**Employment**									
Employed	32 (19.4)	11 (20.4)	21 (19.4)	0 (0.0)	.69	8 (66.7)	24 (21.6)	0 (0.0)	**<.001**
Unemployed	133 (80.6)	43 (79.6)	87 (80.6)	3 (100.0)		4 (33.3)	87 (78.4)	42 (100.0)	
**Smoking history**									
Never smoked	40 (24.2)	12 (22.2)	27 (25.0)	1 (33.3)	.87	8 (66.7)	27 (24.3)	5 (11.9)	**<.001**
Smoked	125 (75.8)	42 (77.8)	81 (75.0)	2 (66.7)		4 (33.3)	84 (75.7)	37 (88.1)	
**IDU history**									
Never IDU	106 (64.2)	35 (64.8)	70 (64.8)	1 (33.3)	.53	12 (100.0)	76 (68.5)	18 (42.9)	**<.001**
IDU	59 (35.8)	19 (35.2)	38 (35.2)	2 (66.7)		0 (0.0)	35 (31.5)	24 (57.1)	
**HIV VL**									
≤ 40	134 (81.2)	44 (81.5)	88 (81.5)	2 (66.7)	.81	12 (100.0)	88 (79.3)	34 (81.0)	.22
>40	31 (18.8)	10 (18.5)	20 (18.5)	1 (33.3)		0 (0.0)	23 (20.7)	8 (19.0)	
**CD4 count**									
≥ 200	155 (93.9)	49 (90.7)	103 (95.4)	3 (100.0)	.46	12 (100.0)	106 (95.5)	37 (88.1)	.15
<200	10 (6.1)	5 (9.3)	5 (4.6)	0 (0.0)		0 (0.0)	5 (4.5)	5 (11.9)	
**HIV infection duration (years)**									
≤ 20	70 (42.4)	26 (48.1)	42 (38.9)	2 (66.7)	.37	8 (66.7)	53 (47.7)	9 (21.4)	**.003**
>20	95 (57.6)	28 (51.9)	66 (61.1)	1 (33.3)		4 (33.3)	58 (52.3)	33 (78.6)	
**Multimorbidity** *									
None	78 (47.3)	24 (44.4)	54 (50.0)	1 (33.3)	.703	9 (75.0)	52 (46.8)	17 (40.5)	.106
≥ 2 comorbidities	87 (52.7)	30 (55.6)	54 (50.0)	2 (66.7)		3 (25.0)	59 (53.2)	25 (59.5)	
**Polypharmacy**									
<10 medications	125 (75.8)	44 (81.5)	80 (74.1)	1 (33.3)	.13	12 (100.0)	88 (79.3)	25 (59.5)	**.005**
≥ 10 medications	40 (24.2)	10 (18.5)	28 (25.9)	2 (66.7)		0 (0.0)	23 (20.7)	17 (40.5)	
**History of falls**									
No	103 (62.4)	38 (70.4)	63 (58.3)	2 (66.7)	.33	11 (91.7)	77 (69.4)	15 (35.7)	**<.001**
Yes	62 (37.6)	16 (29.6)	45 (41.7)	1 (33.3)		1(8.3)	34 (30.6)	27 (64.2)	
**History of poor cognition**									
No	124 (75.2)	44 (81.5)	79 (73.1)	1 (33.3)		11 (91.7)	90 (81.1)	23 (54.8)	
Yes	41(24.8)	10 (18.5)	29 (26.9)	2 (66.7)	.12	1 (8.3)	21 (18.9)	19 (45.2)	**.001**
**History of hepatitis C**									
No	79 (47.9)	25 (46.3)	50 (46.3)	2 (66.7)	.78	12 (100.0)	54 (48.6)	11 (26.2)	**<.001**
Yes	86 (52.1)	29 (53.7)	58 (53.7)	1(33.3)		0 (0.0)	57 (51.4)	31 (73.8)	

**Abbreviations:** FP: Fried Frailty Phenotype; FI: Frailty Index; IDU: Injection Drug Use

* The comorbidities used to define multimorbidity are hypertension, diabetes, hyperlipidemia and GFR <60 ml/min.

**Table 3.** Multivariable Logistic Regression Analysis showing Factors Associated with Frailty using the 40-item CCFI.

Variable	Adjusted Odds Ratio	P value	Confidence Interval
History of poor cognition	3.64	**.003**	1.57-8.46
Multimorbidity (2-3 comorbidities)	1.15	0.76	0.47-2.85
Multimorbidity (>3 comorbidities)	3.06	**.03**	1.13-8.31
History of falls	5.09	**<.001**	2.26-11.52
Polypharmacy (6-9 medications)	3.07	**.03**	1.09-8.63
Polypharmacy (≥ 10 medications)	4.25	**.009**	1.43-12.66
HIV infection duration >20 years	2.67	**0.03**	1.13-6.32
CD4 count <200 cells/ml	3.48	.10	0.78-15.48
HIV RNA >40 copies/ul	.86	.76	0.32-2.24
History of smoking	3.16	**.05**	1.00-9.90
History of IDU	3.17	**.004**	1.44-6.99
History of HCV	2.77	**.01**	1.23-6.25
Thrombocytopenia	5.72	**.001**	2.14-15.29
BMI	1.32	.58	0.51-3.44
Disability	3.80	**.004**	1.52-9.46

Each variable was adjusted for age and sex given at birth.

IDU: Injection Drug Use; HCV: Hepatitis C Virus; BMI: Body Mass Index.
