# Supplementary material for: The Performance of a New Multidimensional Frailty Index in Comparison to the Frailty Phenotype to Assess Frailty in People Living with HIV 50 Years of Age and Older in an Urban HIV Clinic
Source: J AIDS HIV Treat. Author manuscript; Available in PMC 2025 Jun 2. (PMC12129442; doi:10.33696/aids.7.058)
Supplement: JAHT-25-058-Supplementary-Files [file NIHMS2080833-supplement-JAHT-25-058-Supplementary-Files.zip › JAHT-25-058-Supplementary-Files.pdf]

Eke UA, Wasserstein K, Susman C, Eke AC, Mohanty K, Schmalzle S, et al. The Performance of a New Multidimensional Frailty Index in Comparison to the Frailty Phenotype to Assess Frailty in People Living with HIV 50 Years of Age and Older in an Urban HIV Clinic. J AIDS HIV Treat. 2025;7(1):27-37.

Article Title: The Performance of a New Multidimensional Frailty Index in Comparison to the Frailty Phenotype to Assess Frailty in an Urban HIV Clinic.

Supplementary Material

Supplementary Figure 1. Frailty Phenotype Characteristics

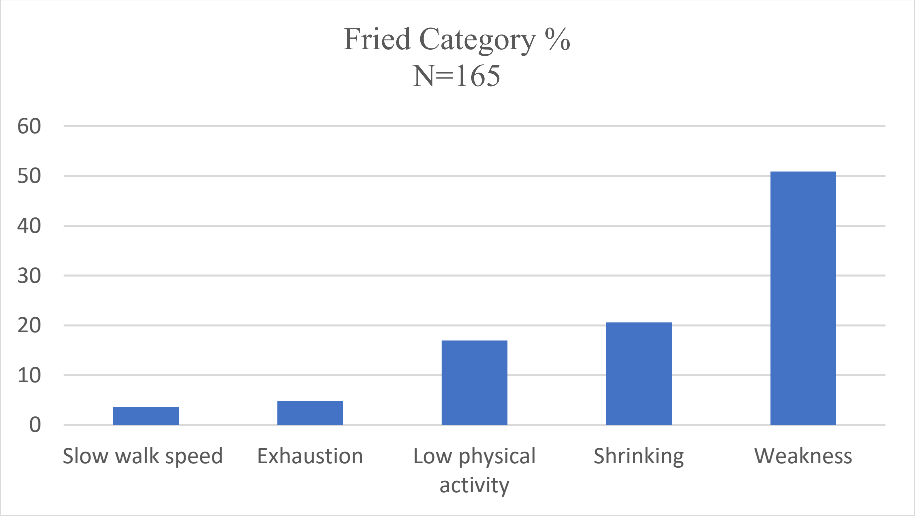

Supplementary Table 1: Multivariable Logistic Regression Analysis showing Factors Associated with Frailty, using a Modified 39-item CCFI Frailty that excludes each specific Factor from the CCFI*.

**Variable**	**Adjusted Odds Ratio**	**P value**	**Confidence Interval**
History of Poor cognition	2.48	**.03**	1.08-5.71
*Multimorbidity (2-3 comorbidities)	1.24	0.64	0.51-2.99
*Multimorbidity (>3 comorbidities)	1.79	0.27	0.64-4.95
History of falls	2.16	**.002**	1.61-7.62
Polypharmacy (6-9 medications)	4.30	**.005**	1.56-11.90
Polypharmacy (>=10 medications)	4.35	**.008**	1.46-12.96
HIV infection duration >20 years	1.63	0.24	0.73-3.65
History of smoking	1.77	.27	0.65-4.81
History of IDU	2.03	.08	.93-4.42
History of HCV	1.77	0.15	0.82-3.87
Thrombocytopenia	3.55	**.01**	1.35-9.32
Disability	2.31	.06	0.97-5.46

Each variable was adjusted for age and sex given at birth.
* Hypertension, diabetes and GFR were excluded from the index for multimorbidity. Hence, a 37-item index was used for multimorbidity.
